# Caspase-9: A Multimodal Therapeutic Target With Diverse Cellular Expression in Human Disease

**DOI:** 10.3389/fphar.2021.701301

**Published:** 2021-07-09

**Authors:** Maria I. Avrutsky, Carol M. Troy

**Affiliations:** ^1^ Department of Pathology and Cell Biology, Vagelos College of Physicians and Surgeons, Columbia University, New York, NY, United States; ^2^ Department of Neurology, Vagelos College of Physicians and Surgeons, Columbia University, New York, NY, United States; ^3^ The Taub Institute for Research on Alzheimer’s Disease and the Aging Brain, Vagelos College of Physicians and Surgeons, Columbia University, New York, NY, United States

**Keywords:** caspase-9, apoptosis, neurodegeneration, retina, cardiomyopathy, innate immunity, cancer, cardiovascular disease

## Abstract

Caspase-9, a cysteine-aspartic protease known for its role as an initiator of intrinsic apoptosis, regulates physiological cell death and pathological tissue degeneration. Its nonapoptotic functions, including regulation of cellular differentiation/maturation, innate immunity, mitochondrial homeostasis, and autophagy, reveal a multimodal landscape of caspase-9 functions in health and disease. Recent work has demonstrated that caspase-9 can drive neurovascular injury through nonapoptotic endothelial cell dysfunction. *CASP9* polymorphisms have been linked with various cancers, neurological disorders, autoimmune pathologies and lumbar disc disease. Clinical reports suggest alterations in caspase-9 expression, activity or function may be associated with acute and chronic neurodegeneration, retinal neuropathy, slow-channel myasthenic syndrome, lumbar disc disease, cardiomyopathies, atherosclerosis and autoimmune disease. Healthy tissues maintain caspase-9 activity at low basal levels, rendering supraphysiological caspase-9 activation a tractable target for therapeutic interventions. Strategies for selective inhibition of caspase-9 include dominant negative caspase-9 mutants and pharmacological inhibitors derived from the XIAP protein, whose Bir3 domain is an endogenous highly selective caspase-9 inhibitor. However, the mechanistic implications of caspase-9 expression and activation remain indeterminate in many pathologies. By assembling clinical reports of caspase-9 genetics, signaling and cellular localization in human tissues, this review identifies gaps between experimental and clinical studies on caspase-9, and presents opportunities for further investigations to examine the consequences of caspase activity in human disease.

## Introduction

Caspases are a conserved family of cysteine-aspartic proteases that mediate programmed cell death and inflammation. Cells express caspases as inactive zymogens, which activate rapidly in response to cellular signals. Initiator caspases (caspase-1, -2, -8, -9, -10) activate through dimerization upon binding to an activating platform. Once active, initiator caspases activate downstream effector caspases (such as caspase-3, -6, and -7) by proteolytic cleavage, which can in turn activate additional initiator caspases (McComb et al., 2019). Consequently, an initiator caspase can trigger a signaling cascade that activates multiple initiator and effector caspases. Caspase cleavage does not degrade substrate proteins, but rather introduces loss-of-function or gain-of-function signaling events (Bratton and Salvesen, 2010). Effector caspases have hundreds of identified cleavage substrates, and the cellular consequence of caspase activity is often a cooperative function of multiple proteolytic events (Julien and Wells, 2017), enabling these proteases to play diverse roles in physiological and pathological contexts.

Here, we focus our attention on clinical reports of altered caspase-9 activity or function in human tissues to provide an overview of the clinical correlates between caspase-9 and disease pathologies. Due to the lack of clinicals approaches for modulating caspase-9 activity, these studies are by necessity correlative. However, functional human genetics and animal models provide mechanistic insight into the role of caspase-9 in disease progression. Our goal is to assemble a framework linking *in vitro* and *in vivo* experimental studies with clinical literature on caspase-9 involvement in human disease.

## Caspase-9 Activity and Regulation

**FIGURE 1:**
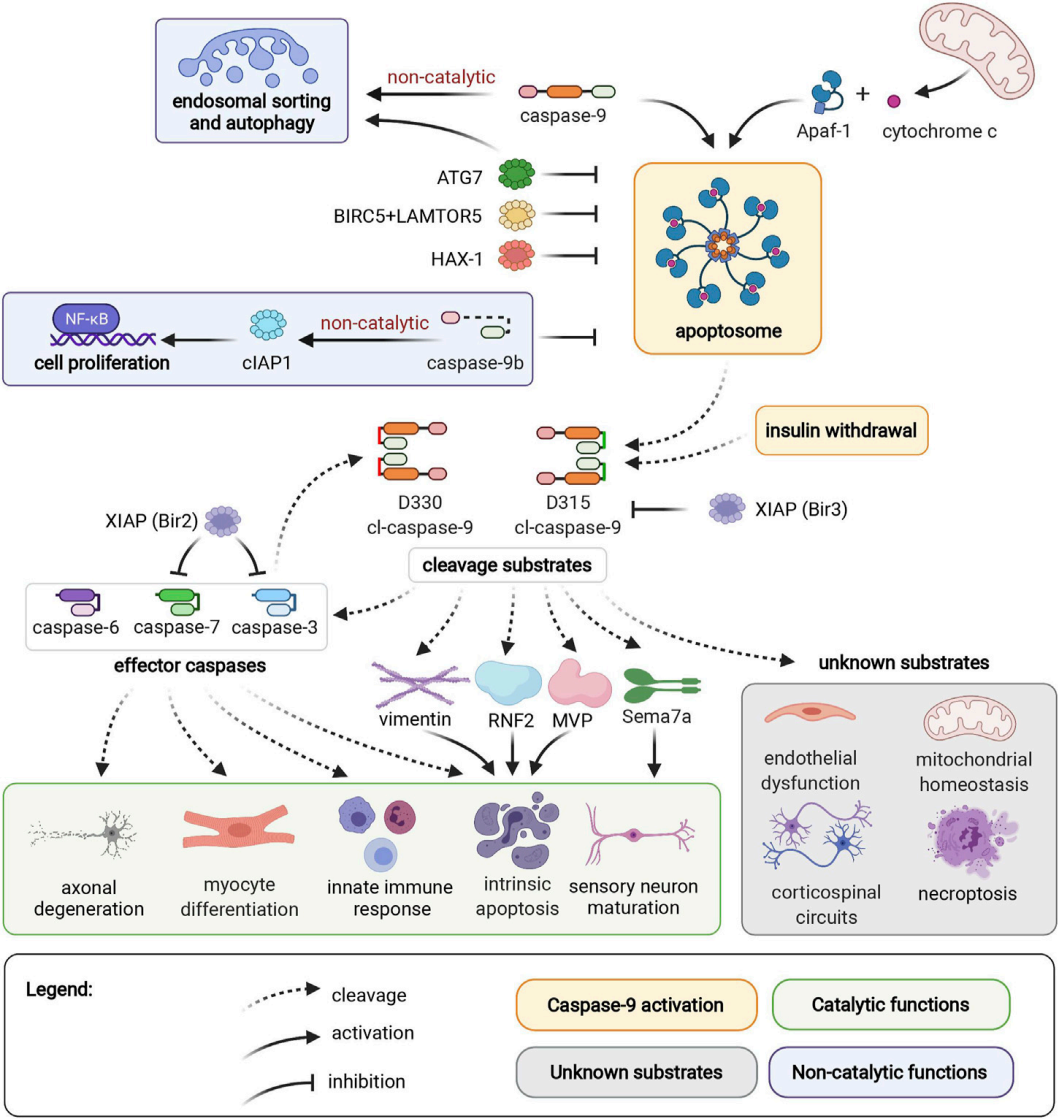
Caspase-9 activation, cleavage substrates, and cellular functions. Caspase-9 can be activated by binding to Apaf-1 in the apoptosome, or by an independent mechanism induced by insulin withdrawal. Endosomal sorting and autophagy are regulated by caspase-9 through a non-catalytic mechanism. ATG7, BIRC5/LAMTOR5 complex, and HAX-1 inhibit caspase-9 activation. Non-catalytic caspase-9b inhibits caspase-9 activation, and promotes cell proliferation through the NF-κB pathway. D315 and D330 neoepitopes of caspase-9 are generated by autocleavage, or caspase-3 cleavage, respectively. Caspase-9 substrates include caspases -3, -6, -7, vimentin, RNF2, MVP, and Sema7a. These cleavage events mediate axonal degeneration, myocyte differentiation, innate immune response, intrinsic apoptosis, and sensory neuron maturation. Caspase-9 mediates endothelial dysfunction, corticospinal circuits, mitochondrial homeostasis, and necroptosis through unidentified mediator substrates. The Bir3 domain of XIAP inhibits D315 cl-caspase-9, while the Bir2 domain of XIAP inhibits effector caspases-3 and -7. Apoptotic protease activating factor 1 (Apaf1); Autophagy Related 7 (ATG7); survivin (BIRC5); Late Endosomal/Lysosomal Adaptor, MAPK And MTOR Activator 5 (LAMTOR5); HCLS1-Associated Protein X-1 (HAX-1); Ring Finger Protein 2 (RNF2); Major Vault Protein (MVP), semaphorin 7a (Sema7a); X-linked Inhibitor of Apoptosis Protein (XIAP).

Caspase-9 activation occurs in the apoptosome, a heptameric structure comprised of Apaf-1 (Apoptotic protease activating factor 1) and cytochrome c, which forms in response to permeabilization of the mitochondrial outer membrane and release of cytochrome c into the cytosol (Figure 1) (Bratton and Salvesen, 2010). Binding to Apaf-1 activates full-length pro-caspase-9 through proximity-induced dimerization and conformational change, which brings its catalytic subunits together to form an active site. An alternative pathway of caspase-9 activation that does not require Apaf-1 has been reported in HeLa cells and in hippocampal neural stem cells upon induction of autophagy by insulin deprivation (An et al., 2019). In both activation modalities, active caspase-9 autocleaves to generate a cleaved caspase-9 (cl-caspase-9) neoepitope at D315. Caspase-9 can also be cleaved by caspase-3, generating an alternate neoepitope at D330. Both D315 cl-caspase-9 and D330 cl-caspase-9 are fully active proteases. However, the two neoepitopes are differentially susceptible to regulation by XIAP (X-linked Inhibitor of Apoptosis Protein), since the Bir3 (Baculoviral Inhibitory Repeat 3) domain of XIAP selectively inhibits caspase-9 at the D315 epitope (Denault et al., 2007). Neoepitope-specific antibodies for the D315 and D330 cleavage sites thus provide insight into caspase signaling pathways, since the D330 neoepitope indicates prevalent co-current caspase-3 activity.

Caspase-9 can activate effector caspases, or act directly on other cellular substrates, by cleaving target proteins after an aspartic acid residue surrounded by a caspase-9 cleavage motif. The most well-characterized consequence of caspase-9 activation is cleavage of downstream effector caspases to induce apoptotic cell death. During apoptosis, caspase-9 helps dismantle intermediate filaments *via* direct cleavage of vimentin (Nakanishi et al., 2001), and inhibits polycomb protein function by cleaving Ring Finger Protein 2 (RNF2) (Wong et al., 2007). Meanwhile, caspase-9 cleavage of caspase-6 contributes to axonal degeneration in ischemic stroke (Akpan et al., 2011). Additional cellular substrates of caspase-9 include Major Vault Protein (MVP), which can be inactivated by caspase-9 cleavage in apoptotic epithelial cells (Grossi et al., 2020). Interestingly, MVP cleavage is observed in primary human keratinocytes and fibroblasts, but not in THP-1 cells (monocytic cell line), highlighting the importance of considering cell-specific contexts when investigating caspase activity. In addition to promoting apoptotic cell death, caspase-9 contributes to necroptosis, an immune-system mediated form of cell death that serves as a backup mechanism to extrinsic cell death. Caspase-9 interacts with necrosis mediator RIPK3 (receptor-interacting serine/threonine-protein kinase 3), and in an *in vivo* model of pancreatic necroptosis, knockout of caspase-9 from pancreatic acinar cells results in decreased severity of cerulein-induced acute pancreatitis (Molnár et al., 2021).

Caspase-9 also has multiple physiological nondeath functions. Caspase-9-mediated activation of effector caspases promotes myocyte differentiation and proliferation (Murray et al., 2008), hematopoietic development (Lu et al., 2014), and immune response to viral infection (Rongvaux et al., 2014). Meanwhile, caspase-9-mediated cleavage of semaphorin7a is required for proper axonal projection during olfactory development (Ohsawa et al., 2010). Intriguingly, both caspase-9 and semaphorin7a (Morote-Garcia et al., 2012; Hu et al., 2018) mediate endothelial hypoxia and inflammatory injury responses. Caspase-9 plays both apoptotic and nonapoptotic roles in ischemic neurovascular injury; ischemic neurons die by caspase-9 mediated apoptosis, while nonlethal activation of caspase-9 in endothelial cells contributes to capillary nonperfusion and vascular edema (Avrutsky et al., 2020). However, it is not known if caspase-9 and semaphorin7a act through shared or separate pathways in endothelial cells. Nonapoptotic caspase-9 signaling is also essential for postnatal motor circuit reorganization, and mice with deficient caspase-9 activation exhibit corticospinal circuit defects and skilled movement deficits (Gu et al., 2017). Corticospinal axon elimination depends on nonapoptotic caspase-9 activity, and this function likely does not require effector caspases-3, -6 or -7 (Gu et al., 2020). Caspase-9 activity is also essential for mitochondrial homeostasis; pharmacological or genetic ablation of caspase-9 results in depolarization of mitochondrial membrane potential, reduced reactive oxygen species production, aberrant accumulation of mitochondrial fusion-fission proteins and reduced autophagy flux (An et al., 2019). This function is dependent on caspase-9 cleavage activity, but it is not clear if effector caspases are relevant substrates for this pathway. Meanwhile, non-catalytic caspase-9 regulates endosomal sorting and lysosomal biogenesis by facilitating retrograde transport of IGFR2 from endosomes to the trans-Golgi network (Han et al., 2020b).

Regulatory pathways enable fine-tuned cellular control of caspase-9 activity. Caspase-9b, an endogenous alternatively-spliced short isoform of caspase-9, lacks the large catalytic subunit and inhibits apoptosis by competing with full-length caspase-9 for binding to the apoptosome (Srinivasula et al., 1999). Additionally, caspase-9b can activate the NF-κB pro-survival pathway through interaction with cIAP1 (cellular Inhibitor of Apoptosis 1) (Srinivasula et al., 1999; Vu et al., 2016). Caspase-9b expression is upregulated in several cancers, potentially providing an apoptosis-evasion strategy for tumors with high levels of caspase-9 expression (Shultz and Chalfant, 2011). Inhibitory phosphorylation (Allan and Clarke, 2009; Serrano et al., 2017) and nitrosylation (Török et al., 2002; Zhang et al., 2016) sites regulate caspase-9 activation by interfering with caspase-9 binding to the apoptosome, or by preventing proteolytic processing of caspase-9 by other caspases (McDonnell et al., 2008). Additionally, antiapoptotic proteins such as XIAP (Denault et al., 2007; Han et al., 2014), survivin/BIRC5 (Baculovirus IAP Repeat Containing 5) complexed with LAMTOR5 (Late Endosomal/Lysosomal Adaptor MAPK and MTOR Activator 5) (Marusawa et al., 2003), HAX-1 (HCLS1-Associated Protein X-1) (Han et al., 2006; Yan et al., 2015), and ATG7 (Autophagy Related 7) (Han et al., 2014) downregulate the apoptotic potential of caspase-9. This milieu of caspase-9 regulation enables a diversity of cell- and context-specific consequences of caspase-9 activation.

## Experimental and Clinical Strategies for Modulating Caspase-9

Genetic approaches enable specific inhibition of caspase-9 activity, either through deletion of the *CASP9* gene, or *via* ectopic expression of catalytically inactive caspase-9 constructs, which function as dominant negative mutants (Lopez-Hernandez et al., 2004; Wang et al., 2011; Baechler et al., 2019). Alternatively, selective pharmacologic inhibition of caspase-9 can be achieved by leveraging cell permeant peptides to enable intracellular delivery of the Bir3 domain of XIAP (Akpan et al., 2011; Avrutsky et al., 2020). Inhibitors based on LEHD, a tetrapeptide caspase inhibitor, are also commonly used to inhibit caspase-9. However, such methods lack specificity due to the overlapping cleavage motifs of apoptotic caspases (McStay et al., 2008; Pereira and Song, 2008). Likewise, cleavage assays based on tetrapeptide substrates (such as DEVD or LEHD) indicate when caspases are active in a tissue sample, but cannot reliably differentiate between specific caspases. The lack of selectivity enabled by these tools results in ambiguous interpretation of data due to the frequent involvement of multiple caspases in converging signaling pathways. Since caspase-9 (and downstream effector caspases) can mediate both apoptotic and nonapoptotic cellular functions, readouts such as TUNEL staining or annexin A5 uptake are useful tools for determining when caspase activity is associated with cell death.

Selective induction of apoptosis *via* inducible caspase-9 has potential therapeutic utility as a safety-switch against overgrowth of cellular grafts. Rivo-cel (CaspaCIDe System, or inducible caspase-9) is a clinical-stage technology developed by Bellicum, which involves pre-transplant transfection of donor T-cells with a chemically inducible caspase-9 gene that can be activated by a small molecule dimerization enhancer (Wu et al., 2014). The therapy entered several phase II trials for improving hematopoietic stem cell transplantation outcomes in the treatment of leukemias, lymphomas and inherited blood disorders by conferring prophylaxis against graft vs host disease. Inducible caspase-9 is also being explored as a potential fail-safe system to protect against the tumorigenic potential of stem cell therapies (Nishimura et al., 2019).

Clinical tools for inhibiting caspase-9 activity are far more limited, as no selective caspase-9 inhibitors have yet entered clinical development. Emricasan [IDN-6556], a small molecule irreversible pan-caspase inhibitor, entered clinical trials for treatment of non-alcoholic steatohepatitis (NASH), portal hypertension, and liver failure/cirrhosis. The treatment was generally well tolerated, supporting the clinical feasibility of targeting caspases as a therapeutic strategy, but failed to meet primary efficacy endpoints (Harrison et al., 2020). The lack of clinical tools for inhibiting caspase-9 activity or expression presents a challenge for interpreting the mechanistic significance of caspase-9 alterations in human disease pathologies. Consequently, our current understanding of the pathologic implications of caspase-9 relies on correlational studies from patient data and, when available, interventional studies in animal models of disease.

## Genetics of Caspase-9 in Human Disease

**FIGURE 2:**
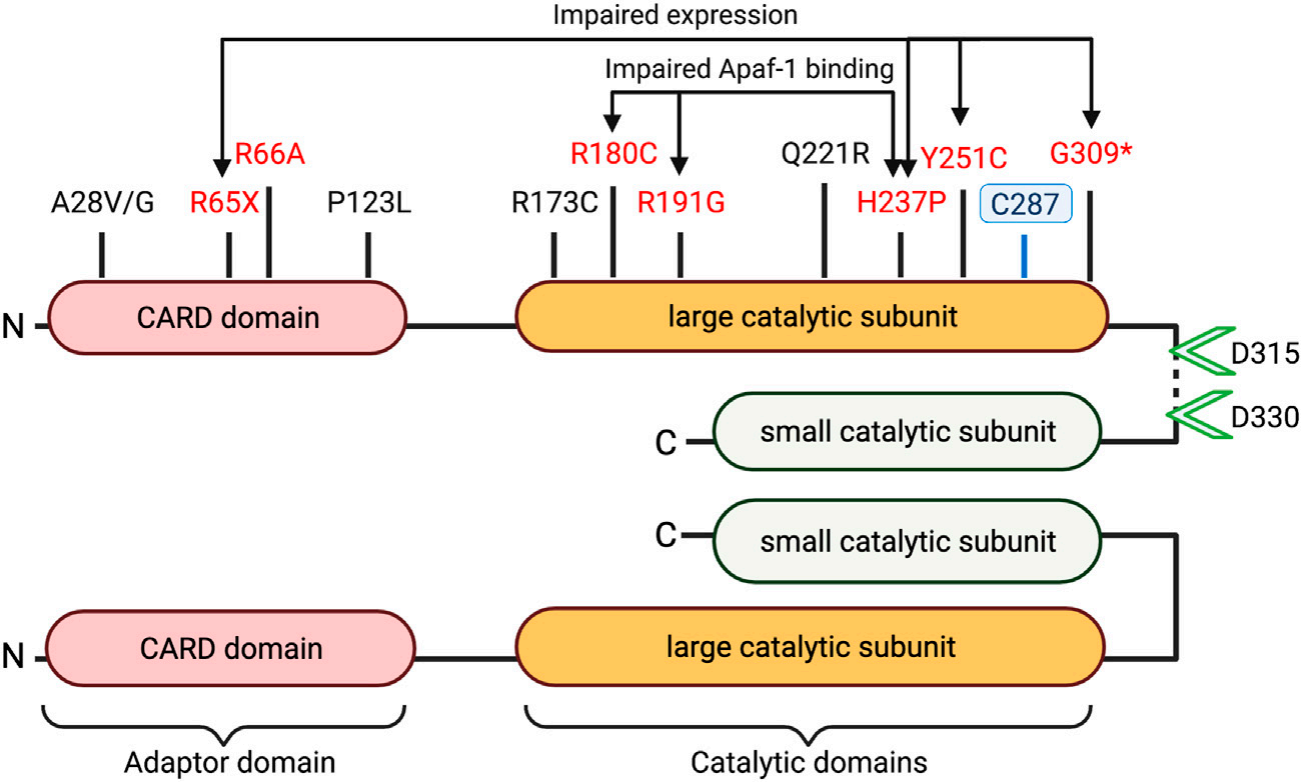
Caspase-9 domains and coding sequence variants. Schematic structure of dimerized active caspase-9 domains is depicted with proteolytic cleavage sites (green arrowheads) and catalytic site cysteine at C287 indicated. Disease associated variants marked in black (uncharacterized mutations) or red (loss of function). (* = Frameshift, X = stop-gain).

Table 1 highlights caspase-9 promoter and gene sequence variants that have been associated with differential health outcomes. Studies have characterized several mutations in the caspase-9 adaptor domain and large catalytic domain, which result in impaired apoptotic potential (Figure 2). Loss-of-function mutations in *CASP9* (as well as in apoptosis-related genes *CASP3* and *APAF1*) have been identified in cases of neural tube defects (NTDs) (Liu et al., 2018; Spellicy et al., 2018; Zhou et al., 2018). Liu et al. (Liu et al., 2018) characterized functional consequences of three variants (G66A, R191G, Y251C) specific to patients with NTDs. All three variants showed impaired apoptosis in 293T cells cultured under low folate conditions, suggesting that folate insufficiency (a known risk factor for NTDs) may be an environmental factor in cases carrying these mutations. The Y251C mutation occurs in a conserved region of the large catalytic subunit, and likely disrupts caspase-9 protein stability; 293T and NE-4C cells express Y251C caspase-9 at 0.35–0.5-fold lower levels compared to WT constructs (Liu et al., 2018; Zhou et al., 2018). The H237P variant (rs146054764), occurring in *trans* with a G309* frameshift mutation in two sibling fetuses diagnosed with craniorachischisis, results in complete absence of detectable caspase-9 protein expression, and substantially downregulated apoptosis in fetal fibroblasts, suggesting that this amino residue is critical for caspase-9 protein stability (Spellicy et al., 2018). Meanwhile, the R180C and R191G variants express at normal levels in transfected 293T cells, but fail to effectively induce apoptosis due to inability to interact with Apaf-1 (Liu et al., 2018; Zhou et al., 2018). Protein prediction analysis indicates that these substitutions may have a damaging consequence on caspase-9 protein structure.

**TABLE 1:** Caspase-9 polymorphisms linked to human disease.

Ensembl	Polymorphism/gnomAD	*CASP9* tissue eQTL	Association (affect allele risk ↑ or ↓)	Ref.
rs4645978	Promoter 1-15852034-C-T	Brain - Spinal cord **↑**	Lumbar disc disease **↓**	Guo et al. (2011b); Zhang et al. (2013a); Mu et al. (2013)
		Mammary tissue **↑**	Breast cancer ↓	Theodoropoulos et al. (2012)
		Whole blood **↑**	Acute myeloid leukemia ↓	Cingeetham et al. (2014)
		Pancreas **↑**	Pancreatic cancer ↓	Theodoropoulos et al. (2010)
		Colon **↑**	Colorectal cancer **↑**	Theodoropoulos et al. (2011)
		Stomach **↑**	Gastric cancer **↑**	Liamarkopoulos et al. (2011)
rs4645981	Promoter 1-15851483-G-A	N/A	Breast cancer **↑**	Lee et al. (2010)
			Lung Cancer **↑**	Yoo et al. (2009); Lee et al. (2010); Xu et al. (2012)
			Hepatocellular carcinoma **↑**	Zhang et al. (2017)
			Acute myeloid leukemia **↑**	Cingeetham et al. (2014)
			General cancer risk **↑**	Zhang et al. (2013b)
rs4645983	Synonymous (S31S) 1-15850603-G-A	Whole Blood ↓ CD4^+^ T cells ↓ Stomach **↑** Small intestine **↑**	Response to corticosteroid or azathioprine for Crohn’s disease ↓	Cravo et al. (2014)
			Sensitivity to dietary fat in Crohn’s disease ↓	Ferreira et al. (2010)
			Response to infliximab for Crohn’s disease **↑**	Hlavaty et al. (2005)
rs4661636	Intron 1-15823061-C-T	Whole blood ↓	Non-Hodgkin’s lymphoma ↓	Lan et al. (2009)
		Esophagus ↓	Esophageal adenocarcinoma **↑**	Liu et al. (2010)
rs2020902	Intron 1-15834360-A-G	Blood ↓	Non-Hodgkin’s lymphoma ↓	Kelly et al. (2010)
		Kidney **↑**	New-onset diabetes after renal transplant **↑**	McCaughan et al. (2014)
rs2042370	Intron 1-15841742-G-A	Whole blood **↑**	Non-Hodgkin’s lymphoma ↓	Kelly et al. (2010)
rs6685648	Intron 1-15825195-T-C	Whole blood **↑**	Non-Hodgkin’s lymphoma **↑**	Kelly et al. (2010)
		Lung **↑**	Lung cancer **↑**	Lin et al. (2012)
rs1052571	Coding (A28V/G) 1-15850613-G-A	Brain **↑**	Ischemic stroke ↓	Lee et al. (2017)
		Whole Blood **↑**	Gentamicin efficacy in Meniere’s disease ↓	Huang et al. (2019)
		Colon **↑**	Severe ulcerative colitis ↓	Guo et al. (2011a)
	Stop-gain R65X		Brain tumors **↑**	Ronellenfitsch et al. (2018)
	Coding (G66A); loss of function		Neural tube defects **↑**	Liu et al. (2018); Zhou et al. (2018)
	Coding (P123L)		Neural tube defects **↑**	Zhou et al. (2018)
rs372045782	Coding (R173C)	N/A	Neural tube defects **↑**	Liu et al. (2018); Zhou et al. (2018)
	Coding (R180C); loss of function		Neural tube defects **↑**	Zhou et al. (2018)
	Coding (R191G); loss of function		Neural tube defects **↑**	Liu et al. (2018); Zhou et al. (2018)
rs1052576	Coding (Q221R) 1-15832543-T-C	Brain - Spinal cord **↑** Brain **↑**	Lumbar disc disease **↑**	Sun et al. (2011)
			Non-Hodgkin’s lymphoma **↑**	Lan et al. (2007)
			Multiple myeloma **↑**	Hosgood et al. (2008)
			General cancer risk **↑**	Zhang et al. (2013b)
			Glioma **↑**	Ozdogan et al. (2017)
			Multiple sclerosis **↑**	Andreoli et al. (2009)
rs146054764	Coding (H237P); dominant negative 1-15832495-T-G	N/A	Immunodeficiency/lymphoproliferation **↑**	Clemente et al. (2015)
			Neural tube defects **↑**	Spellicy et al. (2018)
rs552167727	Coding (Y251C); loss of function	N/A	Neural tube defects **↑**	Liu et al. (2018); Zhou et al. (2018)
	Frameshift (G309*)		Neural tube defects **↑**	Spellicy et al. (2018)

List of disease associations reported for caspase-9 polymorphisms, and associated eQTLs (GTex Portal) indicating whether the SNPs are associated with increased or decreased levels of caspase-9 expression. Reference sequence IDs from dbSNP.

Caspase-9 variants associated with cases of NTDs replicate the phenotype of *CASP9* deletion in murine studies, which results in embryonic lethality due to severe brain malformations and hindbrain neural tube defects (Kuida et al., 1998). Knockout animals exhibit disorganization of cerebral structures, reduced neuronal apoptosis, accumulation of necrotic tissues in the brain, and frequent intracerebral hemorrhages (Hakem et al., 1998), reflecting the importance of caspase-9 in neural development.

Caspase-9 coding variants have also been associated with immune system pathologies. Localized deletion of murine caspase-9 from hematopoietic progenitors impairs leukocyte proliferation and differentiation (Lu et al., 2014), and dysregulates cell intrinsic immune response (Rongvaux et al., 2014). Clemente et al. (Clemente et al., 2015) describes how the H237P (rs146054764) variant may predispose affected individuals to immunodeficiency and lymphoproliferation. Functional characterization of the H237P variant in peripheral blood mononuclear cells (PBMCs) from the affected patients, and in PBMCs from healthy donors transfected with a H237P construct, reveals defective proliferative and apoptotic responses. In transfected 293T cells, H237P caspase-9 fails to associate with Apaf-1, and produces a dominant negative effect on the activation of WT caspase-9. *Ex vivo* analysis of patient lymphocytes and *in vitro* transfection studies show that deficits in caspase-9 activity cause impaired lymphocyte apoptosis and activation *via* downregulation of BAFFR (B-cell-activating factor belonging to the TNF family (BAFF) receptor) in B cells and ICOS (inducible T-cell costimulator) in T cells.

Several other *CASP9* polymorphisms have also been associated with autoimmune and hematopoietic pathologies. The coding variant A28V/G (rs1052571) is associated with lower risk of severe ulcerative colitis (Guo et al., 2011a), and the Q221R (rs1052576) mutation has been identified as a susceptibility locus for multiple sclerosis in Italy (Andreoli et al., 2009). Both loci are associated with significantly increased *CASP9* expression across multiple tissues by eQTL (expression Quantitative Trait Loci) analysis on the GTex Portal. While it has been speculated that the Q221R variant may cause a conformational change modifying the affinity of caspase-9 to Apaf-1 (Xu et al., 2012), the functional consequences of the A28V/G and the Q221R mutations have not been experimentally shown to alter caspase-9 activity or function. Comparison of genotyped primary human lymphocytes found no differences in benzo [a]pyrene-7,8-9,10-diol epoxide (BPDE)-induced apoptosis in cells carrying the Q221R mutation compared to WT caspase-9 (Hu et al., 2008). Thus, functional validation of the A28V/G and Q221R mutations will be necessary to establish if these variants alter caspase-9 activity, its recruitment to the apoptosome, or its expression in a way that contributes to the associated autoimmune phenotypes.

Even greater challenges relate to understanding noncoding *CASP9* polymorphisms associated with hematopoietic malignancies (Hosgood et al., 2008; Lan et al., 2009; Kelly et al., 2010; Cingeetham et al., 2014), new-onset diabetes after renal transplant (McCaughan et al., 2014), and treatment efficacy for Crohn’s disease (Hlavaty et al., 2005; Ferreira et al., 2010; Cravo et al., 2014). Biochemical analysis of patient samples can help identify if these noncoding variants are correlated with alterations in caspase-9 activity or expression in disease-relevant tissues.

### Genetics of Caspase-9 in Human Cancer

Evasion of apoptosis is a hallmark of human cancers, and the role of caspases in carcinogenesis has been studied extensively (Olsson and Zhivotovsky, 2011; Shalini et al., 2015), due to their key functions in regulating cellular differentiation, proliferation, and apoptosis. Caspase-9 can suppress tumor growth by triggering intrinsic apoptosis in response to cellular damage such as genomic instability, oxidative stress, and aberrant proliferation. However, while some tumors inhibit caspase-9 as an apoptosis-evasion strategy (Chee et al., 2013), cellular survival is regulated by interactions of multiple apoptotic proteins. Pharmacological inhibition of caspase-9 can help sensitize cancer cells to chemotherapy by promoting caspase-8-dependent apoptosis (Tamaki et al., 2014). Tumor cells can also hijack caspase-9 signaling to suppress radiation-induced immunity; deletion of caspase-9 or pharmacological pan-caspase inhibition with Emricasan can sensitize cancer cells to radiation therapy by rescuing type I interferon production in irradiated cells (Han et al., 2020a). Additionally, apoptosis not only induces cell death, but also triggers cellular proliferation in surrounding tissues, which can promote tumor growth (Fogarty and Bergmann, 2017).

Mutation or loss of *CASP9* heterozygosity is rarely observed in human cancers (Olsson and Zhivotovsky, 2011; Ronellenfitsch et al., 2018). The strongest functional genetic association between caspase-9 and cancer is a stop-gain mutation at R65, which results in a catalytically inactive short isoform of caspase-9, and may cause increased susceptibility to development of anaplastic astrocytomas consistent with Li-Fraumeni-like syndrome (Ronellenfitsch et al., 2018). A heterozygous germline R65X mutation was identified in two related patients and two unaffected family members. Analysis of the patients’ anaplastic astrocytomas revealed additional tumor-specific mutations in *IDH1* and *TP53*, and absence of caspase-9 immunoreactivity in both tumor and healthy brain tissues. An additional *CASP9* stop-gain mutation was identified in a pediatric glioma patient, suggesting that caspase-9 loss-of-function may contribute to the pathogenesis of some pediatric brain cancers (Muskens et al., 2020).The Q221R mutation of caspase-9 has been associated with increased risk of multiple malignancies (Lan et al., 2007; Hosgood et al., 2008; Zhang et al., 2013b; Ozdogan et al., 2017), however as discussed previously, the functional consequence of this single nucleotide polymorphism (SNP) remains unclear.

Several intron and promoter region polymorphisms in *CASP9* have been associated with incidence and progression of various cancers, including lung (Yoo et al., 2009; Lee et al., 2010; Lin et al., 2012; Xu et al., 2012), esophagus (Liu et al., 2010; Costa et al., 2019), liver (Zhang et al., 2017), breast (Theodoropoulos et al., 2012), and hemopoietic malignancies (Lan et al., 2007; Hosgood et al., 2008; Lan et al., 2009; Kelly et al., 2010; Cingeetham et al., 2014). However, eQTL analysis shows no concordance between tissue-specific effects of disease-associated SNPs on caspase-9 expression, and the directionality of associated disease risks (*see* Table 1). Furthermore, some variants have reported opposite risk effects for different cancers. The *T* allele at the *CASP9* promoter SNP rs4645978 has been associated with increased risks of colorectal (Theodoropoulos et al., 2011) and gastric (Liamarkopoulos et al., 2011) cancers, but decreased risks of breast cancer (Theodoropoulos et al., 2012), acute myeloid leukemia (Cingeetham et al., 2014), and pancreatic cancer (Theodoropoulos et al., 2010). Similarly, the *T* allele of the intron polymorphism rs4661636 is associated with lower risk of non-Hodgkin’s lymphoma (Lan et al., 2009) and higher risk of esophageal adenocarcinoma (Liu et al., 2010). Some cautions should be applied in interpreting conclusions from a single report of an association between a genetic variant and disease susceptibility, with greater weight placed upon associations replicated across multiple study cohorts (Xu et al., 2012; Zhang et al., 2013b; Yan et al., 2013). It should also be emphasized that due to linkage disequilibrium, a disease-associated SNP can tag multiple alleles. Thus, even robust associations between a genetic locus and a disease phenotype requires additional validation to link a specific gene product with a pathogenic SNP. Consequently, while multiple polymorphisms near the *CASP9* locus associate with differential cancer risk, further study will need to clarify which, if any, of these variants cause pathogenic alterations in caspase-9 activity or expression.

## Caspase-9 in Neurologic Pathologies

The most well-studied context of pathological caspase-9 activation is in neurodegenerative disease, where multiple studies have reported increased expression of both total and cl-caspase-9 in apoptotic neurons, glial cells, serum and CSF (Table 2). Neurodegenerative disorders are characterized by progressive loss of neuronal function, and typically feature an increasing burden of neuronal cell death over the course of disease. Treatment strategies for neurodegenerative pathologies typically focus on supportive therapies, and interventions that modify the progression of neuronal loss are still mostly in experimental stages of development (Gan et al., 2018).

**TABLE 2:** Studies reporting increased level of caspase-9 in neurodegenerative pathologies.

Disease (*n*)	Controls (*n*)	Casp9 Measured	Detection Method	Tissue Examined	Pathological Casp9 Localization	Ref.
MSA (10)	Normal (8)	D330 cl-casp9	IHC	Basal ganglia, cerebellum, midbrain, pons, medulla (postmortem)	Oligodendrocytes, neurons, glia	Kawamoto et al. (2016)
ALS (5–8)	Cerebrovascular disease w/o spinal cord pathology (3–5)	Unspecified cl-casp9	IHC	Spinal cord, anterior horn (postmortem)	Motor neurons	Inoue et al. (2003)
		Caspase activity	LEHD-cleavage			
ALS (30)	Tension-type headache (30)	Total casp9	ELISA	Serum	--	Ilzecka (2012)
AD (7)	Normal (7)	D315 cl-casp9	IHC	Hippocampus, frontal cortex (postmortem)	Neurons, neurofibrillary tangles	Rohn et al. (2002)
AD (50) aMCI (50)	Normal (50)	Full-length casp9	Western blot	Blood	Platelet rich plasma	Zhao et al. (2016)
HD (19)	Normal (6)	D330 cl-casp9	Western blot, IHC	Striatum (postmortem)	Neuropil, neurons	Kiechle et al. (2002)
TBI (9)	Normal (5)	Unspecified cl-casp9	ELISA	CSF	--	Darwish and Amiridze (2010)
TBI-severe (45)	Normal (25)	Total casp9	ELISA	CSF	--	Jiang et al. (2020)

MSA, Multiple system atrophy; ALS, Amyotrophic lateral sclerosis; AD, Alzheimer’s disease; aMCI, amnesic mild cognitive impairment; HD, Huntington’s disease; TBI, Traumatic brain injury.

Kiechle et al. (Kiechle et al., 2002) reported increased expression of cl-caspase-9 in postmortem brains from patients with Huntington’s disease (HD), an inherited neurodegenerative disease caused by CAG repeat expansions in the huntingtin gene. HD features neuronal loss in the striatal part of the basal ganglia, leading to movement, cognitive and psychiatric disorders. Brain samples from patients with end-stage disease had greater expression of caspase-3 and D330 cl-caspase-9 in striatal neurons, suggesting activation of the apoptotic cascade. The study found that increasing cytosolic cytochrome c immunoreactivity in neurons was associated with increased striatal degeneration, indicating that activation of intrinsic apoptotic signaling contributes to neuronal death at end-stage disease. Increased caspase activity was also reported in cultured blood cells from HD patients (Squitieri et al., 2011), suggesting that CAG repeat expansions result in increased susceptibility to caspase activation. HD progression has also been associated with mitochondrial dysfunction (Yablonska et al., 2019), potentially implicating both apoptotic and mitochondrial functions of caspase-9 signaling in HD neurodegeneration. Expression of endogenous inhibitor of apoptosis proteins, IAP1 and XIAP, is markedly reduced in HD post-mortem human brain tissue, pointing to IAP-mediated regulation of caspase activity as a potential therapeutic target in HD (Goffredo et al., 2005).

Kawamoto et al. (Kawamoto et al., 2016) examined cl-caspase-9 expression in postmortem brain sections from donors with multiple system atrophy (MSA), a rare sporadic neurodegenerative disorder. The study found D330 cl-caspase-9 in oligodendrocytes and in neuronal and glial cytoplasmic inclusions, supporting involvement of both caspase-9 and caspase-3 in neurodegenerative pathology. Rodent and nonhuman primate models of multiple system atrophy (Lee et al., 2019; Marmion et al., 2021) offer experimental tools to investigate cell-specific consequences of caspase-9 activation in neurons and glia and enable studies to test the therapeutic potential of caspase-9 inhibitors as a neuroprotective strategy in MSA.

Increased caspase-9 activity has also been implicated in Alzheimer’s disease, the most common cause of dementia in older adults, whose neuronal pathology features progressive accumulation of amyloid plaques, neurofibrillary tangles, and synaptic loss. Rohn et al. (Rohn et al., 2002) reported autocleaved (D315) caspase-9 expression in neurons and neurofibrillary tangles in hippocampal sections from patients with Alzheimer’s disease (AD), implicating caspase-9 activation and caspase-3 mediated cleavage of tau in AD pathogenesis. More recently, Zhao et al. (Zhao et al., 2016) found progressively increasing levels of total caspase-9 and other pro-apoptotic proteins in platelet rich plasma from patients with amnesic mild cognitive impairment (a loss of cognitive function that can progress to an AD diagnosis) and AD compared to subjects without cognitive deficits. The correlation between caspase-9 blood protein levels and cognitive impairment suggest systemic alterations in apoptosis regulation may be implicated in the progression of neurodegenerative disease. Both neurovascular pathologies and systemic abnormalities in innate immune system response have been implicated in AD pathogenesis (Fani Maleki and Rivest, 2019). Given the enormous complexity of AD neurodegeneration, it is not surprising that alterations in caspase-9 signaling have been noted across multiple cell types in AD tissues. Cell-type specific inhibition strategies can help discriminate between different caspase-9 signaling pathways and elucidate which caspase-9 activities are potential therapeutic targets in AD.

Two studies reported increased expression of cleaved and total caspase-9 in amyotrophic lateral sclerosis (ALS), a neuromuscular disease driven by degeneration of motor neurons in the brain and spinal cord. Inoue et al. (Inoue et al., 2003) identified cl-caspase-9 in spinal cord motor neurons and measured increased caspase activity by LEHD-cleavage in anterior horn lysates from patients with ALS. The study also showed that overexpression of XIAP in motor neurons (which inhibits caspases-9,-3,-7) attenuated disease progression in a SOD1 transgenic mouse model of ALS. More recently, Ilzecka et al. (Ilzecka, 2012) measured increased levels of total caspase-9 in serum samples from patients with diagnosed ALS. The study found that serum caspase-9 correlated significantly with both duration and severity of ALS. These findings invite further investigation to explore how caspase-9 activity is systemically dysregulated in ALS and to test if pharmacological approaches targeting caspase-9 can replicate the therapeutic benefit of XIAP overexpression.

The association of increased caspase-9 expression with neurodegeneration is not limited to chronic neurodegenerative diseases. Two studies reported increased levels of full-length and cleaved caspase-9 in CSF of patients with traumatic brain injury (TBI) (Darwish and Amiridze, 2010; Jiang et al., 2020). Although these studies do not indicate the cellular origin of CSF caspase-9, immunohistochemical localization data on caspase-7 (a downstream target of caspase-9) in surgical resections of injured brain tissue after severe TBI, suggests involvement of neuronal, glial, perivascular and hematopoietic caspase activation (Zhang et al., 2006). In a rat model of blunt ocular trauma, treatment with a highly selective caspase-9 inhibitor, Pen1-XBir3, reduces neuronal death and improves retinal function, supporting the role of caspase-9 in mediating neuronal death in an acute trauma model (Blanch et al., 2014). Animal models of TBI can help evaluate the therapeutic potential of caspase-9 inhibitors in treating neurovascular and neuroinflammatory sequelae of traumatic brain injury.

Neurodegenerative pathologies features upregulated caspase-9 in neuronal and glial cells, as well as in patient CSF and blood serum samples. These studies indicate that caspase-9 mediates common pathways of neuronal injury across multiple neurodegenerative pathologies, and suggest increasing caspase-9 involvement in later stages of disease when neuronal death is prevalent. However, further study is needed to elucidate how different neurodegenerative etiologies induce caspase-9 activation, to clarify the significance of caspase-9 activity in non-neuronal cell types, and to determine whether targeting caspase-9 activity can offer a therapeutic strategy for treating neurodegenerative disease. Given the multimodal role of caspase-9 in regulating neuronal circuit organization, mitochondrial homeostasis, innate immune response, and neurovascular barrier disruption, it is highly likely that caspase-9 involvement in neurodegenerative pathologies is likewise heterogeneous.

## Caspase-9 in Retinal Neuropathy

Retinal cell death contributes to permanent vision loss in ophthalmic disease, and neuroprotective treatment strategies may translate to vision-saving treatments for retinal disorders such as photoreceptor degeneration, diabetic retinopathy, and glaucoma (Pardue and Allen, 2018). Several studies have implicated caspase-9 in ocular pathologies, supporting caspase-9 as a potential therapeutic in treatment of degenerative eye disease (Table 3).

**TABLE 3:** Studies reporting increased level of caspase-9 in retinal disease.

Disease (*n*)	Controls (*n*)	Casp9 Measured	Detection Method	Tissue Examined	Pathological Casp9 Localization	Ref.
RD (4)	Normal (10)	Unspecified cl-casp9	IHC	Retina (postmortem)	Photoreceptors	Hisatomi et al. (2008)
RRD (33)	--	D315 cl-casp9	Immunoassay	Subretinal fluid	--	Carpineto et al. (2016)
T2D (10)	Normal (10)	Unspecified cl-casp9	IHC	Retina (postmortem)	Ganglion cell layer	Oshitari et al. (2008)
Glaucoma (10–38)	Normal (10–30)	Unspecified cl-casp9	LC-MS/MS, Western blot, IHC	Retina (postmortem)	Ganglion cells	Yang et al. (2011)

RD, Retinal detachment; RRD, rhegmatogenous retinal detachment; T2D, Type 2 diabetes.

Carpineto et al. (Carpineto et al., 2016) detected D315 cl-caspase-9, and other apoptotic signaling proteins, in subretinal fluid from patients with rhegmatogenous retinal detachment (RRD). Rhegmatogenous detachments are caused by a hole or tear in the retina, which allows fluid to accumulate in the subretinal space, requiring emergency treatment by intraocular surgery. The study associated increased levels of cl-caspase-9 with severity and duration of retinal detachment. Retinal detachment uncouples photoreceptors from choroidal circulation, resulting in hypoxic injury and neuronal death. Data on cellular localization of caspase-9 in human retinal detachments is limited to a study from Hisatomi et al. (Hisatomi et al., 2008), who showed colocalization of cl-caspase-9 with TUNEL^+^ photoreceptors in four retinas from donors with retinal detachment. However, it is not clear if degenerating photoreceptors are the primary source of cleaved caspase-9 in subretinal fluid from RRD. Intriguingly, it is endothelial caspase-9 activity that regulates severity of retinal detachment and subsequent photoreceptor injury in mouse studies (Avrutsky et al., 2020). XIAP overexpression confers neuroprotection in rodent and feline injury models (Zadro-Lamoureux et al., 2009; Wassmer et al., 2017), and pharmacological inhibition of caspase-9 by Pen1-XBir3 reduces severity of retinal detachment in mouse retinal vein occlusion (Avrutsky et al., 2020), supporting caspase-9 activity as a potential therapeutic target for retinal detachments. Pharmacological interventions aimed at reducing apoptotic signaling may confer therapeutic benefit in patients awaiting ocular surgery. Such an approach is being tested with an antiapoptotic Fas receptor antagonist (ONL1204), which recently entered clinical development as a complementary therapy for treating RRD (Krishnan et al., 2019). Therapies targeted at caspase-9 signaling may provide an alternative approach for inhibiting detachment-induced photoreceptor injury.

Caspase-9 activation also occurs in retinal ganglion neurons in patients with type 2 diabetes (Oshitari et al., 2008) and in patients with glaucoma (Yang et al., 2011). To date, studies of caspase-9 in ocular disease have focused on photoreceptors and ganglion neurons, and unbiased profiling of caspase-9 signaling may reveal a broader cellular signature of caspase-9 involvement in ocular disease. Present studies support the pro-apoptotic role of caspase-9 as a mediator of neuronal cell death in both cerebral and retinal pathologies, and future interventional studies in animal models of glaucoma and diabetic retinopathy can help discern the therapeutic potential of caspase-9 inhibition for attenuating retinal pathologies.

## Caspase-9 in Lumbar Disc Disease and Myasthenic Disorders

Musculoskeletal conditions are injuries and disorders that affect the body’s bones, joints, and muscles. Caspase-9 upregulation has been associated with the progression of myopathy in slow-channel syndrome (SCS, a myasthenic disorder caused by mutations in acetylcholine receptors) and in intervertebral disk degeneration in lumbar disc disease (LDD) (Table 4).

**TABLE 4:** Studies reporting increased level of caspase-9 in lumbar and myasthenic disorders.

Disease (*n*)	Control (*n*)	Casp9 Measured	Detection Method	Tissue examined	Pathological Casp9 Localization	Ref.
SCS (4)	Normal (4)	Unspecified cl-casp9	IHC	Anconeus muscle (biopsy)	Neuromuscular junction	Vohra et al. (2004)
LDH (84)	--	Total casp9	IHC	Intervertebral disc (biopsy)	Cartilage, fibroblasts, inflammatory cells	Ozevren et al. (2020)

SCS, Slow-channel syndrome; LDH, lumbar disc herniation.

Vohra et al. (Vohra et al., 2004) reported activation of caspase-9 in neuromuscular junctions from four patients with SCS. Activation of caspase-9 was detected in 15–57% of endplates, similar to the proportion of endplates with degenerating mitochondria or vacuoles, and vastly exceeding the number of neuromuscular junctions with nuclear degeneration. The findings suggest that caspase-9 activation plays a prominent role in SCS degenerative processes. Selective caspase-9 inhibition decreases subsynaptic DNA damage, increases endplate area, and improves ultrastructural abnormalities in an SCS transgenic mouse model (Zhu et al., 2014). Currently, the principal treatment modalities for SCS are long-lived open-channel blockers of the acetylcholine receptor ion channel, but expanding the understanding of the mechanisms of neuromuscular transmission, maintenance, and repair may yield novel therapeutic strategies for SCS and related myasthenia disorders (Farmakidis et al., 2018).

A combination of genetic and histologic studies have identified an association between caspase-9 and lumbar disc disease (LDD). Lumbar disk disease occurs when intervertebral disks degenerate, bulge from the bony area of the lower spine, and eventually herniate. The herniated disk can press on nerve roots in the lumbar spine, causing back pain, weakness or numbness. Treatment strategies for LDD depend on severity of disease, ranging from conservative pain management to surgical interventions that excise herniated lumbar segments, and restorative pharmacological treatments are still in early experimental stages of development (Wu et al., 2020).

Guo et al. (Guo et al., 2011b) examined *CASP9* polymorphisms in patients with lower back pain and identified a significant association with the rs4645978 polymorphism in the *CASP9* promoter region. This association has since been replicated in two other studies (Zhang et al., 2013a; Mu et al., 2013). Sun et al. (Sun et al., 2011) reported an additional association between the caspase-9 Q221R mutation (rs1052576) and lumbar disc disease, however both associations have so far only been tested in populations of Chinese ancestry.

Histologic evidence of caspase-9 involvement in LDD comes from a study by Ozevren et al. (Ozevren et al., 2020), who investigated the relationship between caspase-9 expression in discectomy samples and MRI grading of lumbar disc herniation. Biochemical and histological analyses found that increasing inflammation, collagen fiber deterioration, apoptotic process, TNFα and caspase-9 expression were all associated with more severe disc grading based on lumbar spine MRI. Caspase-9 was detected in degenerating cartilage cells, fibroblasts and extravasated inflammatory cells. Discectomy samples with disc protrusion (a more severe grade of disease) showed 2-fold higher levels of caspase activity, compared to samples with disc sequestration (an earlier stage of disc degeneration), suggesting that disc degeneration increases apoptotic processes. The study implicates caspase-9 in LDD progression, and may inform development of restorative treatments aimed at early inflammatory and degenerative disc changes. However, without further experimental evidence, it remains unclear whether caspase-9 activity drives disc degeneration, or whether increased caspase-9 activation is a downstream correlate of tissue injury.

## Caspase-9 in Immune Pathologies

Biochemical evidence links caspase-9 with immunodeficiency, sepsis, and Behcet’s disease (Table 5). Sepsis is a devastating clinical condition characterized by dysregulated host response to infection, which can lead to multi-organ failure and death. Miliaraki et al. (Miliaraki et al., 2021) examined serum caspase levels in patients with sepsis. Serum caspase-9 was significantly related to levels of BIRC5 (inhibitor of caspase-9 activation), and achieved the best receiver operating characteristic curve (AUROC) for predicting mortality and for discriminating between septic patients and either traumatic systemic inflammatory response syndrome (SIRS) or healthy controls. Caspase-9 regulates expression of type 1 interferons, and caspase-9 deficiency is protective against viral lethality in mice (Rongvaux et al., 2014), supporting further studies into whether caspase-9 inhibition may help improve survival in septic patients.

**TABLE 5:** Studies reporting altered levels of caspase-9 in autoimmune disease.

Disease (*n*)	Controls (*n*)	Casp9 Measured	Detection Method	Tissue examined	Pathological Casp9 Localization	Ref.
Sepsis (107)	Normal (89)	Total casp9	ELISA	Serum	--	Miliaraki et al. (2021)
	SIRS (75)					
Immunodeficiency/lymphoproliferation (2)	--	Caspase activity	LEHD cleavage	Blood	PBMC	Clemente et al. (2015)
		Casp9 H237P	Genotyping			
Behçet’s disease (15)	Normal (9)	Total casp9	IHC	Aphthous lesion (biopsy)	Endothelial cells	Oztas et al. (2007)
Behcet’s disease (7)	Normal (7)	Caspase activity	LEHD cleavage	Blood	Neutrophils	Nazıroğlu et al. (2014)

SIRS, Traumatic Systemic Inflammatory Syndrome; PBMCs, Peripheral blood mononuclear cells.

Increased caspase-9 expression has also been reported in Behcet’s disease, a multisystem disorder of unknown etiology that is driven by endothelial inflammation. Oztaş et al. (Oztas et al., 2007) measured approximately 50% increase in mean caspase-9-positive endothelial cell counts in oral biopsies of aphthous lesions from patients with Behcet’s disease, compared to skin biopsies from healthy controls or patients with psoriasis. Higher levels of caspase activity were also measured in neutrophils isolated from Behcet’s patients (approximately 60% higher than control), along with other markers of apoptosis and oxidative stress (Nazıroğlu et al., 2014). Together these studies associate elevated caspase-9 activity with systemic inflammatory pathologies. While no animal models fully recapitulate the phenotype of Behcet’s disease, several approaches enable relevant experimental study of autoimmune dysregulation (Charles et al., 2020). Since caspase-9 can contribute to both apoptotic and nonapoptotic endothelial dysfunction, examining regulation of caspase-9 activity in different vascular pathologies may give insight into endothelial physiology and vascular health. Likewise, given the association between caspase-9 loss-of-function mutations and immune system deficits (as described in Section 4) further study is warranted to clarify the consequences of altered caspase-9 expression in patient leukocytes and to distinguish between apoptotic and nonapoptotic roles of caspase-9 in immune disease.

## Caspase-9 in Cardiovascular Pathologies

Cardiovascular disorders, including atherosclerosis and cardiomyopathies are the leading causes of mortality in the industrialized world (Arnett et al., 2019). Cardiovascular disease progression culminates in end-stage heart failure, requiring transplantation as the treatment modality of choice in eligible patients. There is an urgent need to develop therapies that can prevent heart failure, and evidence-based treatment algorithms to manage treatment of the disease (Crespo-Leiro et al., 2018).

Atherosclerosis is a disease of the arteries characterized by endothelial activation, inflammation and deposition of fibrous fatty deposits in the vessel walls. Increased expression and activation of caspase-9 has been reported in two studies of atherosclerotic arteries (Table 6). Niculescu et al. (Niculescu et al., 2004) immunostained atherosclerotic human aorta samples, and detected D330 cl-caspase-9 in endothelial cells and in fibrous plaques. Parallel TUNEL staining experiments revealed prevalent cell death in these types of lesions. More recently, Sobenin et al. (Sobenin et al., 2015) measured 2-fold increased expression of *CASP9* mRNA in atherosclerotic fatty streak (type II) lesions compared to unaffected intima, initial lesions, lipofibrous plaques or fibrous plaques from thoracic aorta biopsies. Additionally, the A28V/G mutation in caspase-9 has been associated with decreased risk of ischemic stroke (Lee et al., 2017), supporting a possible causative connection between caspase-9 and vascular disease. These studies provide a glimpse into mechanisms of both transcriptional and post-translational caspase-9 regulation in vascular pathologies. Broad spectrum caspase inhibition reduces cell death in the arteries of rabbits fed a high-cholesterol diet and subjected to aortic balloon de-endothelialization (Sarai et al., 2007), providing supporting evidence that caspases contribute to vascular injury. However the specific role of caspase-9 in atherosclerotic vessels remains oblique, and further study of caspase-9 signaling is warranted in animal models that can recapitulate the lipoprotein metabolism and chronic inflammatory features of atherosclerotic vessels. Dysregulation of innate immunity is a driver of cardiovascular disease (Saltiel and Olefsky, 2017), implying that both pro-apoptotic and immune-regulatory functions of caspase-9 may be involved. Further research may identify common caspase-9-mediated pathways in autoimmune dysfunction and cardiovascular disease.

**TABLE 6:** Studies reporting increased level of caspase-9 in cardiovascular pathologies.

Disease (*n*)	Controls (*n*)	Casp9 Measured	Detection Method	Tissue Examined	Pathological Casp9 Localization	Ref.
ICM (24) CR (15)	Cardiac allograft donors (5)	D315 cl-casp9	Western blot	Myocardium (heart transplant)	--	Tung et al. (2003)
DCM (13)	Aortic stenosis (12)	D315 cl-casp9	Immunoassay	Myocardium (biopsy)	--	Neidhardt et al. (2019)
	ICM (13)					
DCM (36)	Donor hearts (10)	D315 cl-casp9	Western blot, cDNA array	Left ventricle (biopsy)	--	Aharinejad et al. (2008)
		*CASP9* mRNA				
iDCM (22)	DCM (10)	Total casp9	ELISA	Serum, endomyocardium (biopsy)	--	Bironaite et al. (2015)
CP/Rep +	Normal (8)	D330 cl-casp9	Western blot	Right atrium (biopsy)	--	Feng et al. (2013)
Diabetes (16)						
Cardioplegia (30)	Preoperative samples	*CASP9* mRNA	RT-PCR	Blood	--	Elcik et al. (2021)
Atherosclerosis (25)	--	*CASP9* mRNA	RT-PCR	Thoracic aorta (biopsy)	Fatty streak lesions	Sobenin et al. (2015)
Atherosclerosis (12)	Normal (6)	D330 cl-casp9	IHC	Aorta (biopsy)	Endothelium, fibrous plaques	Niculescu et al. (2004)

ICM, Ischemic cardiomyopathy; CR, chronic rejection; DCM, Dilated cardiomyopathy; iDCM, inflammatory dilated cardiomyopathy; CP/Rep, Cardioplegic arrest and reperfusion.

Cardiac pathologies are associated with inflammation arising from innate immune system activation related to cardiac damage, creating a rich milieu of both inflammatory and apoptotic caspase signaling (Jaén et al., 2020). In mouse development, caspase-9 plays an essential nondeath role in muscle and cardiomyocyte differentiation and proliferation (Murray et al., 2008; Bulatovic et al., 2015), supporting both apoptotic and nonapoptotic pathways of potential caspase-9 involvement in heart disease. Biopsy and serum samples from several cardiac pathologies feature increased levels of total and cleaved caspase-9 (Table 6). Differential expression of caspase-9 neoepitopes and downstream effector caspases in different forms of cardiomyopathy suggests that multiple caspase-9 signaling pathways are involved in these disorders.

Two studies identified increased expression of caspase-9 in myocardial tissues following induction of cardioplegic arrest, a technique which induces cessation of cardiac function to minimize myocardial damage during open heart surgeries. Cardioplegic arrest and reperfusion (CR/Rep) renders the heart globally ischemic, and can trigger myocardial injury upon reperfusion. Feng et al. (Feng et al., 2013) showed increased D330 cl-caspase-9 expression and increased TUNEL staining in atrium biopsies collected from diabetic patients after CP/Rep, compared to nondiabetic controls. Combined with the reported increase in caspase-9 activation in diabetic retinas (Oshitari et al., 2008), these results support a common association between diabetes, caspase-9 activation, and increased susceptibility to ischemic cell death across different organ systems. More recently, Elcik et al. (Elcik et al., 2021) showed greater *CASP9* mRNA induction in blood samples from patients who underwent custodiol cardioplegia compared to blood cardioplegia. In combination with other autophagy, apoptosis and hypoxia markers, *CASP9* expression was associated with hypoxic myocardial damage. However, it is not clear how myocardial injury would be mechanistically linked with the reported changes in blood mRNA measures.

Tung et al. (Tung et al., 2003) examined the role of caspase-9 signaling in myocardium and coronary arteries in explanted hearts from patients with ischemic cardiomyopathy (ICM) and patients with chronic rejection of heart transplant. TUNEL labeling of dying myocytes in both ICM and chronic rejection tissues correlated positively with levels of cl-caspase-9 and cl-caspase-3. Cleaved caspase-9 also showed a strong negative correlation to the time of survival of the initial allograft for retransplanted patients. In contrast, a recent study (Neidhardt et al., 2019) examined myocardial biopsies from patients with ischemic cardiomyopathy, and did not detect increased expression of either cl-caspase-9, cl-caspase-3, or cl-caspase-8 compared to aortic stenosis controls. It remains to be clarified whether patient selection or choice of controls substantially influenced the apparent differences in cl-caspase-9 detection in ICM.

Caspase-9 involvement has also been reported in nonischemic myocardial pathologies. Three studies have implicated caspase-9 in dilated cardiomyopathy (DCM), a form of myocardial degeneration that causes weakening and enlargement of the heart. Aharinejad et al. (Aharinejad et al., 2008) reported increased levels of cleaved and total caspase-9 in left ventricle samples from patients with DCM. A later study (Bironaite et al., 2015) surveyed caspase-9 expression in patients with inflammation-positive DCM and inflammation-negative DCM, and found higher levels of caspase-9 compared to caspase-8 or caspase-3, with differential expression in serum and endomyocardial biopsy samples. Most recently, Neidhardt et al. (Neidhardt et al., 2019) reported a 2-fold increase of D315 cl-caspase-9 in myocardial biopsies from patients with DCM and did not detect increased cl-caspase-3 or cl-caspase-8 expression. Together these studies show that specifically caspase-9, but not caspases-3, or -8 are induced in DCM pathology. Selective upregulation of specific caspases in DCM provides a unique opportunity to understand differential signaling roles of these caspases in degenerative and inflammatory pathologies.

To date, studies of caspase-9 involvement in heart disease have relied primarily on biochemical detection methods, and we still do not know the cellular localization of caspase-9 in these pathologies, limiting our understanding of the signaling pathways regulating caspase-9 in cardiomyopathy. These reports remain agnostic regarding whether caspase signaling mediates cell death in DCM patients. On the other hand, CP/Rep and chronic rejection of heart transplant clearly link caspase-9 activation with cell death. Correlational studies indicate a close association between caspase-9 expression and disease severity, however further interventional studies in animal models of DCM would be helpful to establish whether inhibiting caspase-9 may provide therapeutic benefit in these disorders.

## Conclusion

Caspase-9 has multiple apoptotic and nonapoptotic cellular functions, which complicates interpretations of caspase-9 expression in disease tissues. While colocalization of caspase-9 with dying neurons has been established in several neurodegenerative conditions, its nonapoptotic roles in immune regulation, endothelial function, mitochondrial homeostasis and neuronal circuit organization substantially expand the potential scope of caspase-9 involvement in disease (Table 7). In particular, our mechanistic understanding of caspase-9 signaling in lumbar disc disease, cardiovascular disease, and Behcet’s disease are limited to phenomenological observations. Studies can utilize inducible cell-specific knockout of caspase-9 to dissect cellular signaling pathways in the context of animal disease models. Meanwhile, development and adoption of selective inhibitors in experimental models is essential to clarify which caspase signaling pathways are plausible therapeutic targets.

**TABLE 7:** Summary of caspase-9 involvement in human degenerative pathologies.

Disease	Tissue localization of pathologic Caspase-9 expression	Fluid Samples with Increased Caspase-9	Strength of evidence	Summary of evidence linking caspase-9 with human pathology
**Human pathologies associated with Caspase-9 loss of function**				
Immunodeficiency/lymphoproliferation	--	--	Strong	—Functional human genetics
				—Knockout mouse phenotype
Neural tube defects	--	--	Strong	—Functional human genetics
				—Knockout mouse phenotype
Pediatric brain tumors (Li-Fraumeni-like syndrome)	--	--	Strong	—Functional human genetics
**Human pathologies associated with increased Caspase-9 activity or expression**				
Amyotrophic lateral sclerosis	Motor neurons	Serum	Moderate	—Therapeutic effect in animal models
				—Correlation with disease severity
Retinal detachment	Photoreceptors	Subretinal fluid	Moderate	—Therapeutic effect in animal models
				—Correlation with disease severity
Slow-Channel syndrome	Neuromuscular junctions		Moderate	—Therapeutic effect in animal models
				—Increased signal in patient samples
Alzheimer’s disease	Neurons, neurofibrillary tangles	Platelet rich plasma	Weak	—Correlation with disease severity
Atherosclerosis	Aorta	--	Weak	—Increased signal in patient samples
Behcet’s disease	Endothelial cells, neutrophils	--	Weak	—Increased signal in patient samples
Cardiomyopathy	Myocardium	Serum	Weak	—Increased signal in patient samples
Cardioplegia	Myocardium	Blood	Weak	—Increased signal in patient samples
Chronic rejection of heart transplant	Myocardium	--	Weak	—Correlation with disease severity
Diabetic retinopathy	Retinal ganglion neurons	--	Weak	—Increased signal in patient samples
Glaucoma	Retinal ganglion neurons	--	Weak	—Increased signal in patient samples
Huntington’s disease	Neurons, neuropil	CSF	Weak	—Correlation with disease severity
Multiple system atrophy	Neurons, oligodendrocytes, glia	--	Weak	—Increased signal in patient samples
Sepsis	--	Serum	Weak	—Correlation with disease severity
Traumatic brain injury	--	CSF	Weak	—Correlation with disease severity
Lumbar disc disease	Cartilage, fibroblasts, inflammatory cells	--	Weak	—Correlation with disease severity
				—Genetic association studies

Summary of pathologies associated with either caspase-9 loss of function or upregulated caspase-9, and level of evidence implicating caspase-9 as a potential driver of disease pathology.

Human genetic evidence implicates caspase-9 loss of function mutations with neural tube defects, pediatric tumors, and immunodeficiency/lymphoproliferation. These loss of function phenotypes help elucidate the diverse roles played by caspase-9 in different tissues, but are not readily tractable for caspase-9-targeted therapeutic interventions due to the inherent risks of induced cell death caused by caspase-9 upregulation. Gene association studies suggest potential caspase-9 involvement with multiple cancers, autoimmune disorders, and neurological disease. Clinicogenomic analysis of caspase-9 activity and expression in patient tissues should help clarify IF putative pathogenic *CASP9* SNPs are specifically associated with alterations in caspase-9 signaling. Furthermore, coding variants A28V/G (rs1052571) and Q221R (rs1052576) are still not characterized for how they may impact caspase-9 signaling, despite extensive associations of these mutants with multiple cancers and degenerative and inflammatory pathologies. Understanding the functional consequence of these mutations would provide key genetic evidence to determine if caspase-9 is a disease-causing target in diseases associated with these variants.

Pathogenic expression and activation of caspase-9 has been reported in neuronal, vascular, lumbar, myocardial and hematopoietic tissues. Interventional studies in animal models support caspase-9 as a driver of disease pathology in ALS, retinal detachment, and SCS, suggesting that inhibiting caspase-9 activity can be a viable therapeutic strategy for these disorders. However, for the majority of human pathologies associated with upregulated caspase-9, the therapeutic potential of caspase-9 inhibition strategies has not yet been tested. Exploring caspase signaling across different organ systems can help expand our understanding of degenerative diseases by examining cell-type specific dysfunction subsequent to caspase-9 activation. Eye research is readily translatable to CNS disease due to the shared neurovascular architecture of brain and retinal tissues (London et al., 2013), and caspase-9 likely shares signaling pathways in CNS and retinal neurodegeneration. Meanwhile the nonapoptotic role of caspase-9 in immune response may illuminate the pathogenic roles of caspases in cardiovascular, musculoskeletal, and neurodegenerative pathologies.
